# Planum temporale asymmetry in people who stutter

**DOI:** 10.1016/j.jfludis.2017.06.003

**Published:** 2018-03

**Authors:** Patricia M. Gough, Emily L. Connally, Peter Howell, David Ward, Jennifer Chesters, Kate E. Watkins

**Affiliations:** a Department of Psychology, Maynooth University, Maynooth, Co. Kildare, Ireland; b Department of Experimental Psychology, University of Oxford, South Parks Road, Oxford, OX1 3UD, UK; c Department of Psychology, University College London, Bedford Way, London, WC1E 6BT, UK; d School of Psychology and Clinical Language Sciences, University of Reading, Harry Pitt Building, Earley Gate, Reading, RG6 7BE, UK

## Abstract

**Purpose:**

Previous studies have reported that the planum temporale – a language-related structure that normally shows a leftward asymmetry – had reduced asymmetry in people who stutter (PWS) and reversed asymmetry in those with severe stuttering. These findings are consistent with the theory that altered language lateralization may be a cause or consequence of stuttering. Here, we re-examined these findings in a larger sample of PWS.

**Methods:**

We evaluated planum temporale asymmetry in structural MRI scans obtained from 67 PWS and 63 age-matched controls using: 1) manual measurements of the surface area; 2) voxel-based morphometry to automatically calculate grey matter density. We examined the influences of gender, age, and stuttering severity on planum temporale asymmetry.

**Results:**

The size of the planum temporale and its asymmetry were not different in PWS compared with Controls using either the manual or the automated method. Both groups showed a significant leftwards asymmetry on average (about one-third of PWS and Controls showed rightward asymmetry). Importantly, and contrary to previous reports, the degree of asymmetry was not related to stuttering severity. In the manual measurements, women who stutter had a tendency towards rightwards asymmetry but men who stutter showed the same degree of leftwards asymmetry as male Controls. In the automated measurements, Controls showed a significant increase in leftwards asymmetry with age but this relationship was not observed in PWS.

**Conclusions:**

We conclude that reduced planum temporale asymmetry is not a prominent feature of the brain in PWS and that the asymmetry is unrelated to stuttering severity.

## Highlights

• Planum temporale asymmetry was compared in 67 people who stutter and 63 age-matched controls.
• Size or asymmetry of the planum temporale did not differ between people who stutter and controls.
• The asymmetry of the planum temporale was not affected by stuttering severity.
• Differences in asymmetry of the planum temporale are not a cause, consequence or correlate of developmental stuttering.

## 1 Introduction

Historically, altered language dominance has been considered a cause of developmental stuttering. This idea was expressed in the “Cerebral Dominance Theory” that dates back to Orton and Travis (Orton, 1928, Travis, 1931). Recently, brain-imaging studies indicating reduced functional lateralization during speech processing in people who stutter (PWS) lent support to this theory (see meta-analyses by Belyk, Kraft, & Brown, 2015; Budde, Barron, & Fox, 2014; Brown, Ingham, Ingham, Laird, & Fox, 2005). One language-related structure that typically shows leftwards asymmetry is the planum temporale (PT) in the posterior temporal lobe. The relevance of the PT to the altered laterality theory of stuttering arises from the suggestion that the PT is larger and its asymmetry reduced in PWS (Foundas et al., 2001Foundas, Bollich, Corey, Hurley, & Heilman, 2001) and has atypical rightwards asymmetry in those with severe stuttering (Foundas et al., 2004).

**Fig. 1:**
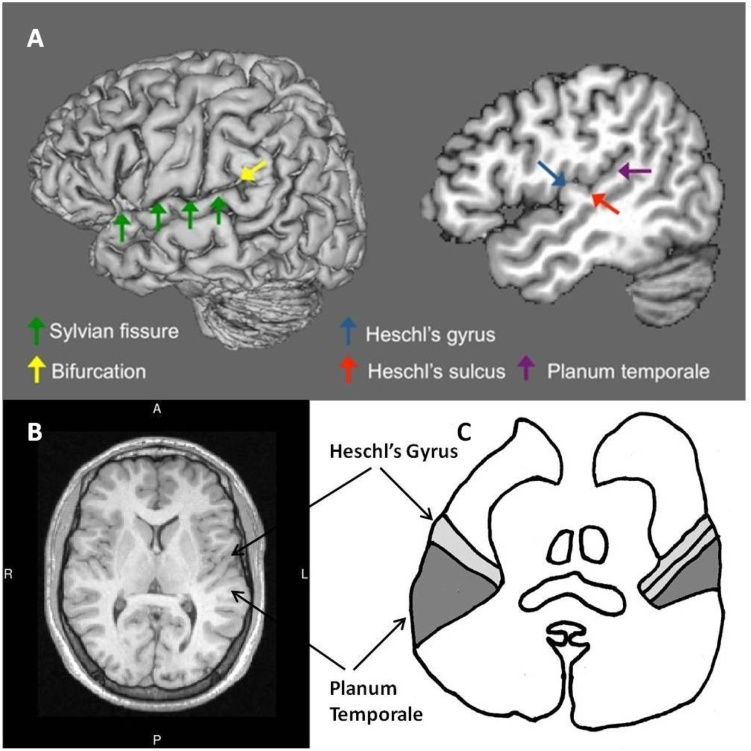
Location and shape of the Planum Temporale (PT). (A) Lateral surface view (left) of the left hemisphere indicating the Sylvian fissure (green arrows) and the bifurcation at its posterior limit into ascending and descending rami (yellow arrow). Sagittal slice (right) through the left hemisphere at 48 mm from the midline indicating Heschl’s gyrus (blue arrow), Heschl’s sulcus (red arrow), and the PT (purple arrow). (B) Axial slice from a structural scan for one participant in this study. A – anterior, P – posterior, R – right, L – left. (C) Simplified line drawing based on Geschwind & Levitsky’s (1968) figure showing the location of Heschl’s gyrus and the PT. (For interpretation of the references to colour in this figure legend, the reader is referred to the web version of this article.)

The PT is located on the horizontal surface of the superior temporal gyrus and extends to the first transverse gyrus (Heschl’s gyrus) anteriorly (Pfeifer, 1936), to the insula medially, and posteriorly to the bifurcation of the Sylvian fissure into the posterior ascending and descending rami (e.g. Foundas et al., 2001, Foundas et al., 2004) (see Fig. 1). When viewed from the superior surface of the temporal lobe, the PT has the appearance of a triangle with its longest side at the lateral extent and its “tip” located medially (see Fig. 1C). The PT is considered to be secondary auditory cortex and is thought to be important for speech and complex sound processing (Binder, Frost, Hammeke, Rao, & Cox, 1996; Caplan, Gow, & Makris, 1995; Griffiths & Warren, 2002) and auditory-motor integration (Hickok, Okada, & Serences, 2009).

In the typically developed brain, asymmetry between the left and right PT is a robust observation − the left side being larger. This was first described based on post-mortem examinations of brains (Geschwind & Levitsky, 1968) and has been confirmed by others using manual and automated measurements of MRI data (e.g. Foundas, Leonard, Gilmore, Fennell, & Heilman, 1994; Watkins et al., 2001). Because of its spatial proximity to the receptive language centre in “classic” neurological models of language in the left hemisphere (‘Wernicke’s area’), the asymmetry of the PT has been taken as a proxy for functional lateralization of language. It was suggested that manual measurement of PT could result in a spurious leftwards asymmetry because of interhemispheric differences in the shape of the posterior Sylvian fissure (Binder et al., 1996) . However, this would not account for differences between PWS and Controls since the same measurement approach was used in both groups (Foundas et al., 2001, Foundas et al., 2004). The finding of reduced overall leftwards asymmetry in stuttering (Foundas et al., 2001) and a group of people with severe stuttering showing rightwards asymmetry (Foundas et al., 2004) ties in with the suggestion that the left hemisphere dominance for language processing that is seen in the vast majority of fluent participants, is not true for PWS. This might further suggest that such a lack of (or reduction in) left hemisphere dominance is at the root of fluency difficulties. As discussed previously (e.g. Packman and Onslow, 2002, Watkins et al., 2015) it is not possible to confirm whether this reduced or reversed asymmetry is a cause, consequence, or correlate of developmental stuttering until longitudinal studies have addressed this question.

It is possible, however, that the findings and conclusions regarding the PT in PWS may be premature. Whereas there was a significant reduction in the leftwards PT asymmetry in a group of 16 PWS relative to 16 Controls (Foundas et al., 2001), the same methods applied to a smaller group of 14 PWS by the same authors failed to replicate this difference in asymmetry between the two groups (Foundas et al., 2004). Furthermore, the reversed (rightwards) asymmetry that reported for cases with severe stuttering was based on a small number of PWS (5 of the 14 PWS studied) and an equal number of participants in the fluent control group (5/14) had reversed asymmetry as well. This makes it difficult to conclude that rightwards asymmetry is unusual (it occurred in 10/28 people studied) or related to dysfluency (it occurred equally often in fluent speakers and PWS). Furthermore, the inclusion of left-handers and females in these studies may have influenced the results since a finding of atypical PT asymmetry has not been replicated in studies that only included right-handed male PWS (Cykowski et al., 2008; Chang, Erickson, Ambrose, Hasegawa-Johnson & Ludlow, 2008).

### 1.1 The current study

Here, we sought to re-examine the question of asymmetry of the PT in PWS. Over a series of studies, we have obtained whole-brain structural images of a large number of PWS. The pooled data from a large sample of 130 individuals provides an opportunity to assess whether the PT asymmetry is altered in PWS compared to controls. The surface area of the left and right-hemisphere PT was measured manually following Foundas et al.’s (2004) procedures. These data were then analysed to assess asymmetry for the two groups. Voxel-based morphometry was also used to provide an automated measure of asymmetry (Watkins et al., 2001). If a reduction in, or lack of asymmetry of, the PT is found in PWS, then this would support an altered language dominance view of developmental stuttering.

## 2 Methods

### 2.1 Participants

There were 130 participants in total, 67 (13 females, 54 males) were PWS and 63 (16 females, 47 males) were Controls, who did not stutter. Out of the 130 participants, 13 were left-handed: nine in the PWS group (five female, four male) and four in the Control group (all male). The mean age of the PWS was 25.90 years (SD = 10.96; range 12–54 years) and of the Controls was 25.25 years (SD = 11.10; range 13–53 years). The groups were well matched for age and years spent in education (neither differed significantly between groups).

Severity of stuttering was determined in the PWS using the most recent version of the Stuttering Severity Instrument (Riley, 1994, Riley, 2009), which varied depending on when the data were obtained (note, however, that the calculation of the stuttering severity score is unchanged across versions; Howell, 2013). Video recordings were used by trained researchers (two were qualified speech and language therapists) to calculate the stuttering severity score; a subset of these videos were re-analysed by two other researchers to ensure accuracy and consistency. The mean total SSI score for the PWS group was 23.63 (SD = 9.22; range 7–43). Stuttering ranged from very mild to very severe. Eleven participants (16.4%) were rated as having a very mild stutter, 21 (31.3%) a mild stutter, 19 (28.7%) a moderate stutter, 10 (14.9%) a severe stutter, and 6 (9%) a very severe stutter. All participants in the PWS group reported that they had stuttered since childhood and had received therapy, though the amount of therapy received varied considerably across the group. All participants spoke English competently though for one participant this was not his native language. One participant identified as a simultaneous bilingual and three others reported speaking or being exposed to another language or dialect since early childhood. However, we did not routinely ask our participants about their language experience and other languages learnt or spoken, so we do not have complete records regarding the degree of bilingualism in our participants (both PWS and Controls). Control participants reported no history of stuttering and were confirmed as having normally fluent speech by the researchers involved in the study.

None of the participants reported any other diagnoses such as dyslexia or impairment in language or learning. None reported any history of neurological disorder. All participants completed a consent form before taking part in the imaging study. The data were collected across several different imaging studies each of which received ethical approval from local or national research ethics committees.

### 2.2 MRI scan acquisition

Structural scans of the whole head were acquired using four different Siemens imaging systems (a 1.5T Sonata, a 1.5T Avanto and two 3T Trios); data from PWS and Controls were acquired on each system. All images were T1-weighted high-resolution (1 mm^3^ voxels) images acquired using either a FLASH or an MPRAGE sequence. Thirty-nine PWS and 38 Controls were scanned at 1.5T (FLASH sequence, single-channel headcoil: TR = 12 ms, TE = 5.65 ms, flip angle = 19^0^; MPRAGE sequence, 12-channel headcoil: TR = 2730 ms, TE = 3.57 ms, flip angle = 7^0^). Twenty-eight PWS and 25 Controls were scanned at 3T (MPRAGE sequence 12-channel headcoil: TR = 2020 ms, TE = 2.9 ms, flip angle = 9^0^; 32-channel headcoil: TR = 2040 ms, TE = 4.7 ms, flip angle = 8^0^). The magnet strength, sequence, and headcoil were thought unlikely to affect the manual measurements as these required simple visualization of the sulcal morphology. It is also unlikely that these factors would differentially affect the two hemispheres of the brain, making the quantitative measurements of asymmetry robust to changes in these acquisition features.

### 2.3 Definition and manual measurement of the PT

For the manual measurement of surface area of the PT, whole-head images were first registered to the MNI-152 standard space brain image using a linear transformation run automatically using FLIRT (https://fsl.fmrib.ox.ac.uk/fsl/fslwiki/FLIRT; 6 degrees of freedom: 3 translations and 3 rotations in x, y, and z axes respectively). This ensured that images were all aligned to the same axes but were not scaled in size. In order to blind the rater measuring the PT, scans were coded without reference to group membership, handedness, or sex. In addition, random subsets of data acquired from each of the four scanners were flipped so that right and left hemispheres were reversed, ensuring measurements were also made blind to hemisphere. Visualisation and measurement of the surface of the PT was performed using FSLview (http://www.fmrib.ox.ac.uk/fsl). Measurement was performed in the sagittal plane while coronal and axial views were used to assist in location of landmarks where necessary.

#### 2.3.1 The Planum Temporale

We followed methods previously described (Foundas et al., 2004, Leonard et al., 1993) tostandardise the measurement and number of sagittal slices included in the PT mask by employing a proportional grid in standard space (Talairach and Tournoux, 1988) . The x-coordinates of the midsagittal plane and the left-most and right-most lateral sagittal slices were identified first with the head rotated to be in standard space and aligned to the MNI152 brain (see above). This allowed the calculation of the width of each hemisphere, each of which was divided into four quadrants. Each quadrant is a standard unit and the PT between 2.25 and 3.25 units was measured in each brain. This method avoids ambiguity in more lateral slices and also ensures that comparable regions are examined across brains (Leonard et al., 1993).

#### 2.3.2 Measurement of surface area of the PT

**Fig. 2:**
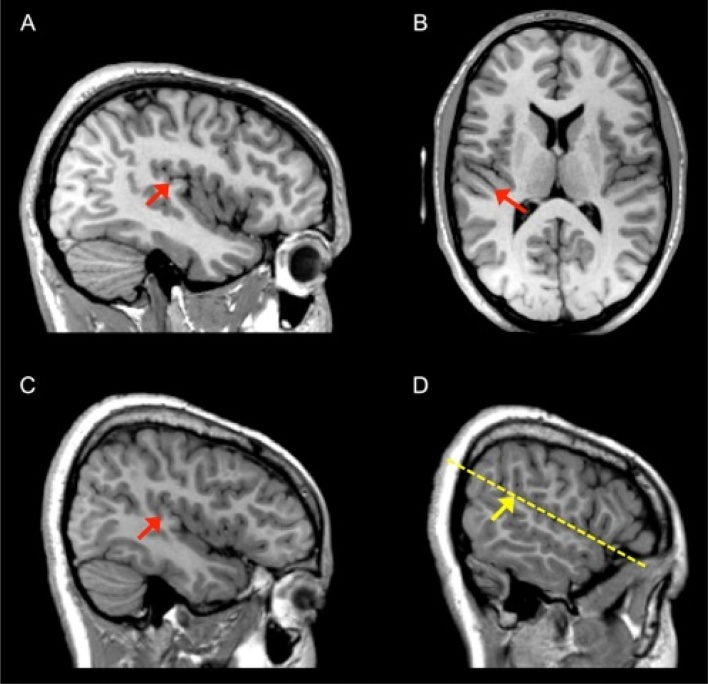
Determining the posterior and anterior boundaries of the PT. (A). A case of a posterior duplication of Heschl’s gyrus. Heschl’s sulcus is indicated by the red arrow on the sagittal slice 39 mm from the midline. (B). Axial slice showing the duplication of Heschl’s gyrus in the same person. (C). A case of a heart-shaped Heschl’s gyrus with an incomplete duplication showing the “common stem” formation. Heschl’s sulcus is indicated by the red arrow on a sagittal slice 40 mm from the midline. (D). Knife-cut method of determining the posterior boundary in the absence of a descending ramus. A sagittal slice 51 mm from the midline is shown. The dotted yellow line indicates the trajectory of a plane through the Sylvian fissure and the yellow arrow indicates the point where this line intersects the parietal wall and the posterior boundary. (For interpretation of the references to colour in this figure legend, the reader is referred to the web version of this article.)

Using FSLview, the surface of the PT was traced for each sagittal slice from the anterior to the posterior boundary (Fig. 2). In cases where Heschl’s gyrus was completely duplicated, the anterior border of the PT was taken as posterior to the first gyrus (i.e. the first Heschl’s sulcus) and the measurement included the second gyrus (see Fig. 2A; Leonard et al., 1993). In cases where the first Heschl’s gyrus appeared heart-shaped when viewed sagittally, and duplication was incomplete (common stem; see Fig. 2C), the gyrus was treated as singular and not included in the PT measurement.

Variability can also occur in the location of the posterior border of the PT. In “classical” patterns of anatomy the bifurcation of the Sylvian fissure provides an obvious locus for the posterior border (see Fig. 1A), however in some scans there was no obvious posterior ascending ramus. In these cases, the “knife-cut” method was employed (Westbury, Zatorre, & Evans, 1999). This approach requires imagining a plane running in line with the Sylvian fissure extending backwards until it intersects with the parietal wall. This intersection point is then taken as the most posterior point of the PT (see Fig. 2D).

Once the surface of the PT had been labelled on the set of contiguous sagittal images in each hemisphere, the number of voxels in the label was calculated to give the total surface area. Following these calculations, data were unblinded and assigned to the correct hemisphere. For each participant, an asymmetry quotient (AQ) was calculated:

AQ = [left PT area − right PT area]/[0.5(left PT area + right PT area)].

### 2.4 Automated calculation of asymmetry using voxel-based morphometry

Even though the manual measurements were made blind to group status, and hemisphere, there is a subjective element. Voxel-based morphometry (VBM) is an automated method for analysis of brain structure. In a previous study, we used VBM to detect structural asymmetries in the brains of 142 neurotypical adults (Watkins et al., 2001). In that study, this method confirmed the normal population asymmetry of the PT among other robust cerebral asymmetries. The same automated approach was used here to reanalyse all images and to examine the asymmetry of the PT in PWS and Controls. Analysis was carried out using tools in the FMRIB Software Library (FSL). Images were first processed using the FSL VBM toolbox (http://fsl.fmrib.ox.ac.uk/fsl/fslwiki/FSLVBM). All steps were run automatically using the scripts in this toolbox and the recommended defaults; the resulting images were checked manually after each step and before analysis. Each whole-head image was first skull-stripped and segmented to form an image of grey matter. These images were registered nonlinearly to the MNI152 template to transform them into standard space. A study-specific template was created, which comprised the nonlinearly registered grey matter images from 63 PWS and 63 Controls (this ensures equal representation of the two groups in the template; four datasets from the PWS were excluded randomly) and their mirror images. The mirror images were created by flipping them across the midline. Averaging the original and the flipped images ensured that the study-specific template was symmetric and that no asymmetry in the template was introduced into the study images. The individual grey matter images of 67 PWS and 63 Controls were then re-registered nonlinearly to the symmetric study-specific template and the signal in each voxel was modulated by the amount to which it had been deformed to match the template. This modulation ensures that the signal in the registered images for each individual represents the original grey matter signal at that location but also compensates for the enlargement or contraction of the original image during the nonlinear transformation. The modulated registered grey-matter images were smoothed using a ∼10-mm full-width at half maximum Gaussian smoothing kernel. The choice of smoothing kernel is consistent with previous studies using this method (e.g. Watkins et al., 2001). Smoothing weights the signal at each voxel according to the signal in the neighbouring voxels, thereby reflecting the regional amount or density or concentration of grey matter (note that this is sometimes referred to as grey matter volume). This process also limits the effects of differences between hemispheres and between individuals in the precise location of anatomical landmarks. In order to evaluate inter-hemispheric differences within participants, a mirror-image of the brain was created for by flipping the image across the midline so that the original left hemisphere was in the place of the original right hemisphere and *vice versa*. Each mirror image was then subtracted from its original counterpart to create an image reflecting the difference in the amount of grey matter between spatially homologous voxels in the two hemispheres. Positive values in the left hemisphere indicated a left-greater-than-right asymmetry and positive values in the right hemisphere indicated a right-greater-than-left asymmetry (negative values reflect the opposite direction asymmetry). A *t*-test at every voxel then compared the groups of PWS and Controls to determine regions where the extent of asymmetry was significantly different. The images were corrected voxel-wise across the whole brain for multiple comparisons using permutation testing implemented in Randomise (http://fsl.fmrib.ox.ac.uk/fsl/fslwiki/Randomise). Voxel values were extracted from the peak of significant difference between the two hemispheres in the PT (the standard space coordinates for this peak in each hemisphere were ± 40, −36, 18) and used for off-line analyses comparing gender, age, and stuttering severity. The PT was identified anatomically in the template using the landmarks described in Section 2.3. The VBM method normalises for brain volume differences, and so rather than analyse a ratio of the left-right difference to the sum of left + right, we simply analysed the difference (the same results were obtained when the ratio was analysed).

## 3 Results

### 3.1 Manual measurement of the PT

#### 3.1.1 Does asymmetry of the PT differ between PWS and Controls?

**Fig. 3:**
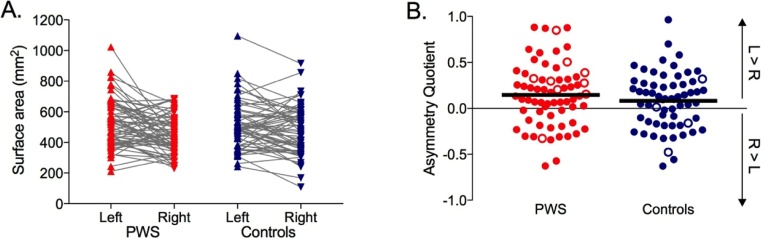
Surface area measurements of the PT. (A). Left and right hemisphere measurements shown for individual PWS and Controls. (B). Asymmetry Quotients for individual participants. Red symbols – PWS, blue symbols – Controls, filled circles – right handers, open circles – left handers, solid black lines – group means, L – left, R – right. (For interpretation of the references to colour in this figure legend, the reader is referred to the web version of this article.)

Analysis of variance with a within-subject factor of hemisphere (left vs. right) and a between-subjects factor of group (PWS vs. Controls) confirmed a significant left-greater-than-right asymmetry in the surface area of the PT (F(1,128) = 15.36, *p <* 0.0005, *η^2^* = 0.11). This significant difference between hemispheres did not differ between groups (the interaction between hemisphere and group was not significant, *p* = 0.120). The overall size of the PT did not differ between the two groups in either hemisphere (no significant main effect of group, *p* = 0.723) (Fig. 3A). Calculating the AQ takes into account individual differences in brain size by expressing the difference between hemispheres as a ratio of the sum of the two hemispheres. The mean AQ across all participants was significantly greater than zero (mean = 0.115 SD = 0.32; one-sample *t*-test t(129) = 4.11, *p <* 0.0005; Fig. 3B) confirming a leftwards asymmetry for this structure; the AQ was not significantly different between PWS and Controls (independent samples *t*-test, *p* = 0.247) and did not correlate significantly with age (*p* = 0.534). Separate correlations did not reveal any significant relationship between PT surface area asymmetry and age in either group (PWS, *p* = 0.696; Controls, *p* = 0.658). Similarly, a regression analysis to determine if there was a significant interaction between age and group revealed that the slopes of the two regression lines were not significantly different (F < 1, *p* = 0.947).

Thirteen of the 130 participants reported being left handed (using their left hand to write; open circles in Fig. 3B). We excluded these participants and re-ran the above analyses. The same pattern of results was observed with a small reduction in effect size in the ANOVA for the significant main effect of hemisphere (*η^2^* *=* 0.10) and a small reduction in the size of the AQ (mean = 0.108, SD = 0.32). It is worth noting that the mean AQ found here is identical to that reported in the original study by Foundas (Foundas et al., 2001).

#### 3.1.2 Does asymmetry of the PT differ between males and females who stutter?

**Fig. 4:**
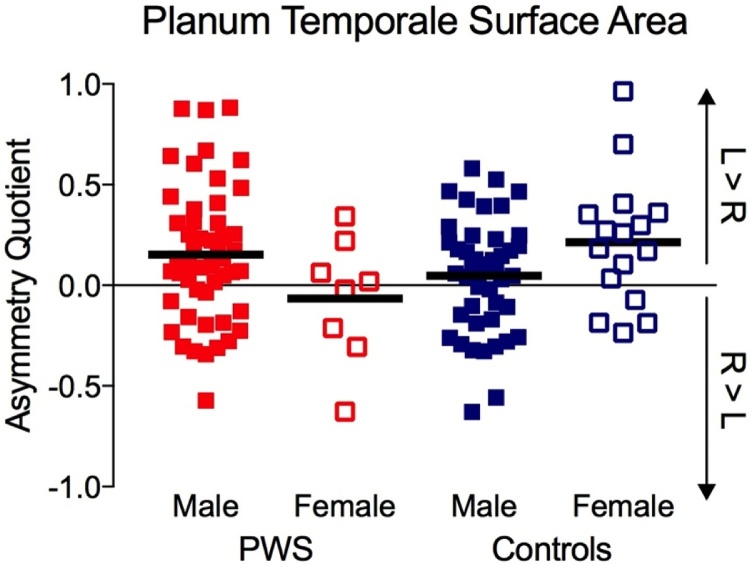
Gender differences in PT surface area asymmetry. Red symbols – PWS, blue symbols – Controls, filled symbols – male, open symbols – female, solid lines – group means. (For interpretation of the references to colour in this figure legend, the reader is referred to the web version of this article.)

Next, the effect of gender on asymmetry of the PT was examined. We carried out a 2 × 2 ANOVA on AQ with between-subjects factors of groups (PWS vs. Controls) and gender (males vs. females). Only data from the right-handed participants (N = 117) were analysed because there were no left-handed, female control participants. There was a significant interaction between group and gender (F(1,113) = 6.64, *p* = 0.011, *η^2^* *=* 0.05) with a notably very small effect size. There were no significant differences between groups (*p* *=* 0.251) and genders (*p* = 0.730). The significant interaction was due to a slightly rightwards AQ for the small group of female PWS but this difference was not quite significant compared with the AQ for the female controls (*p* = 0.058) and the AQs for the two male groups did not differ significantly either (*p* = 0.102) (see Fig. 4).

#### 3.1.3 Does asymmetry of the PT differ in PWS with severe stuttering?

**Fig. 5:**
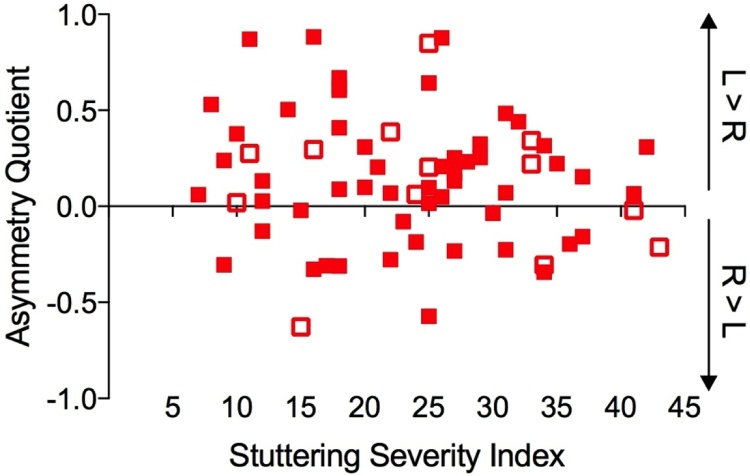
The (lack of) relationship between stuttering severity and asymmetry of the PT. Open symbols – females who stutter, filled symbols – males who stutter, R – Right, L – Left.

Previously, the asymmetry of the PT was reported to be rightwards in the PWS with the highest stuttering severity (Foundas et al., 2004). Therefore, the correlation between AQ and the SSI was examined in our larger sample. There was no significant relationship between PT asymmetry and stuttering severity for the whole group of males and females who stutter (N = 67; *p* = 0.254) nor when only males or only right-handed males were considered (Fig. 5). Nevertheless it is worth observing that the AQ for those with the highest stuttering severity (SSI score >30) is typically between +0.5 and −0.5 (i.e. closer to symmetry) whereas the range of AQs in those with lower stuttering severity scores is much wider.

### 3.2 Voxel-based morphometry (VBM) asymmetry analyses

#### 3.2.1 Does asymmetry of the PT measured using VBM differ between PWS and controls?

**Fig. 6:**
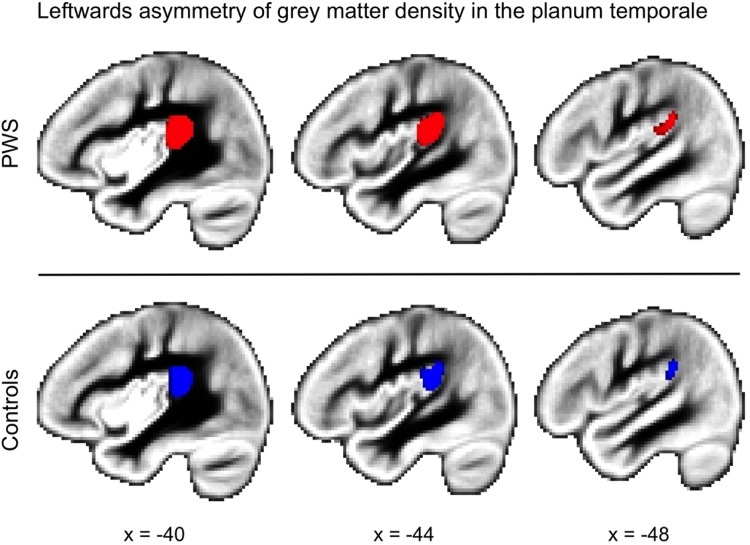
Left-greater-than-right asymmetry of grey matter density in the PT. The brain image is the study-specific template of grey matter density averaged from 63 PWS and 63 Controls. Coloured areas indicate a significant asymmetry in the amount of grey matter (voxel-wise statistics, p < 0.05 corrected using permutation testing) in the PT of the left hemisphere. Red – PWS, Blue – Controls. Sagittal slices are shown through the left hemisphere at 40, 44 and 48 mm from the mid-sagittal plane. (For interpretation of the references to colour in this figure legend, the reader is referred to the web version of this article.)

The automated analysis of asymmetry revealed a significant leftwards asymmetry of the PT in both PWS and Controls (see Fig. 6). Although beyond the focus of this paper, another notable asymmetry observed in both groups was a right-greater-than-left asymmetry of the superior temporal sulcus (see also Watkins et al., 2001). However, the whole-brain analyses revealed no significant differences in asymmetry between the two groups in any brain area following correction for multiple comparisons. Even at an uncorrected statistical threshold of *p <* 0.05, there were no significant group differences in the left-greater-than-right asymmetry of the PT. The location of the peak of the significant PT asymmetry collapsed across both groups was ±40, −36, 18 in MNI-152 standard space. The grey-matter densities for the two hemispheres and the difference between them were extracted from this peak and used for further analyses.

**Fig. 7:**
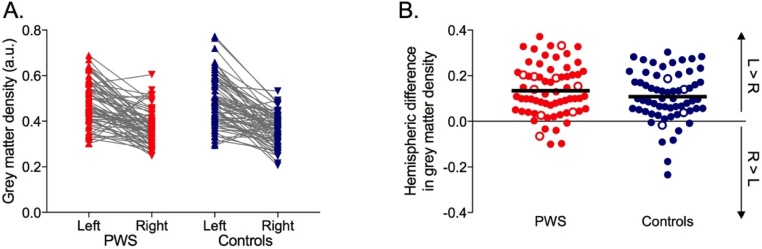
Grey matter density estimates for the PT obtained using voxel-based morphometry (standard space location ±40, −36, 18). See legend of Fig. 3 for details.

To allow comparison of the VBM data with the surface area manual measurements, we have plotted the VBM data in Fig. 7.

**Fig. 8:**
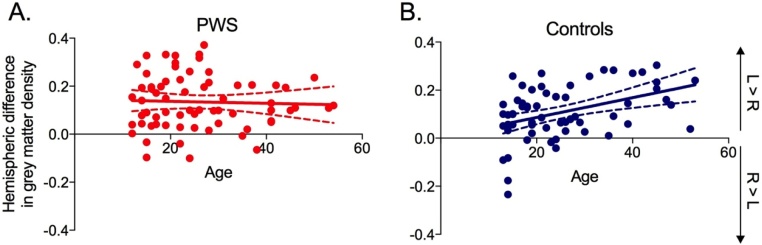
The relationship between age and asymmetry of grey matter density in the PT. This was significant in B. Controls (blue) but not in A. PWS (red). R – Right, L – Left. Solid lines represent the regression line with 95% confidence intervals shown by the dashed lines. (For interpretation of the references to colour in this figure legend, the reader is referred to the web version of this article.)

In contrast to the results of analysis of surface area PT asymmetry above, the analysis of PT asymmetry in grey-matter density revealed a weak but statistically significant correlation with age (Pearson’s r(130) = 0.182; *p* = 0.038) when considering the whole sample. The relationship with age was examined further to ascertain whether it applied to both groups; although there was a significant medium correlation with age and hemispheric difference in PT grey matter in Controls (Pearson’s r(63) = 0.414, *p <* 0.001), there was no relationship in PWS (*p* = 0.748). Linear regression analysis showed that the slopes of the two regression lines differed significantly as well (F(1, 126) = 6.91, *p* *=* 0.010; see Fig. 8). This confirmed a significantly different relationship between age and PT asymmetry in the two groups. Examination of Fig. 8 suggests that the significant relationship of asymmetry with age in Controls might be driven by a small group of young controls with rightwards asymmetry. These participants were scanned on a 1.5T system with an MPRAGE sequence. Even though it is unclear why this particular scanner or sequence would affect a measure of difference between the two hemispheres, we re-analysed the data without these participants and the relationship with age remained significant for Controls and non-significant for PWS. Exclusion of the 13 left-handed participants also did not change the pattern of results.

**Fig. 9:**
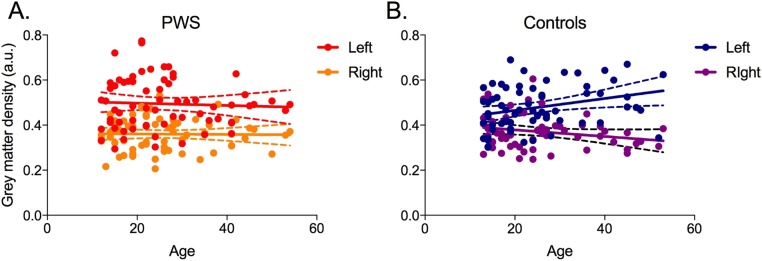
The relationship between age and grey matter density in the left and right PT. This was significant for the left PT in B. Controls (blue) but not in A. PWS (red).Grey matter density in the right PT showed no significant relationship with age in either group (PWS – orange; Controls – purple). Solid lines represent the regression line with 95% confidence intervals shown by the dashed lines. (For interpretation of the references to colour in this figure legend, the reader is referred to the web version of this article.)

To explore the change with age in PT asymmetry for grey matter density, we examined the correlations within each hemisphere separately for the two groups. In Controls, there was a significant positive correlation between age and grey matter density in the left (Pearson’s r(63) = 0.298, *p* = 0.018) but not the right PT (*p* = 0.092). In PWS, neither correlation was significant (left, *p* = 0.688; right, *p* = 0.901). Examination of the data (Fig. 9) reveals that there was a significant age-related increase in grey matter density in the left PT in Controls that was not evident in PWS. The difference between the slopes of the two regression lines for age and left PT grey matter density in Controls compared with PWS was not quite significant (F(1,126) = 3.95, *p* = 0.061).

#### 3.2.2 Does the hemispheric difference in grey matter in the PT differ between males and females who stutter?

Analysis of the right-handed participants revealed that the effect of gender on the left-right difference measure was significant (F(1,113) = 4.69, *p* = 0.032, *η^2^* *=* 0.04) and was attributable to a lower asymmetry in females than males (as noted above in Section 3.1.2 only right-handed participants were included in this analysis as there are no female controls who are left handed to allow a comparison with our left-handed females who stutter). As reported above for the analysis of PT surface area asymmetry (see Section 3.1.2), the size of the effect of gender was very small. Here, the interaction with group was not quite significant (*p* = 0.064) although the pattern of results was similar to that seen for the surface area measurements (Fig. 4).

#### 3.2.3 Is the hemispheric difference in grey matter in the PT related to stuttering severity in PWS?

As in the manual analyses (see Fig. 5), there was no significant relationship between stuttering severity and the hemispheric difference in the amount of grey matter in the PT (*p* = 0.643; data not plotted). The result of the correlation analysis remained non-significant even when left-handed individuals and females were excluded. Further analyses of the relationship of the left and right hemisphere values for the PT with stuttering severity were also not significant (*p* = 0.269 and 0.313 for left and right hemispheres, respectively).

## 4 Summary and discussion of findings

In sum, no evidence was found that the size of the PT or its asymmetry, measured either manually or by automated methods, differed between groups of PWS and Controls. This pattern of results held when the analysis was restricted to only right-handed participants or only male right-handed participants. We conclude, therefore, that atypical asymmetry of the PT is not a feature of the stuttering brain. It should be noted that in previous work (Foundas et al., 2004), similar conclusions were reached regarding a lack of significant difference at the population level in PT asymmetry.

### 4.1 The influence of gender on PT asymmetry

Although the sample size in the current study was large, the number of female PWS was relatively small. Nevertheless, an analysis of the effects of gender revealed that the gender differences in asymmetry differed across PWS and Controls (see Fig. 4). This was a small effect based on a very small number of right-handed females who stutter (N = 8) so should be treated with caution. Furthermore, the effect was not replicated in the automated analysis, where a small but significant overall difference was found between males and females (women had a lower difference in grey matter between the two hemispheres than men, i.e. they were more symmetric) but this pattern was observed in both PWS and Controls.

### 4.2 The influence of age on asymmetry

Use of a large sample size afforded the opportunity of examining the relationship between the PT asymmetry and age. Again, results of different analyses were mixed. For the manual surface area measurement, no relationship was found between the asymmetry and age in either group. Yet, in the automated measurement of grey matter density, the Controls showed a significant increase in PT asymmetry with age that was not evident in the PWS. Furthermore, this relationship between PT asymmetry and age in Controls was driven by an age-related increase in grey matter density in the left PT with no significant change in the right PT; the age-related increase in grey matter density was not evident in either hemisphere in PWS. Different relationships between age and brain measurements were reported previously in another large imaging study of stuttering, which overall found no differences in the grey matter in a group of 55 PWS compared with 61 Controls, aged between 6 and 48 years (Beal et al., 2015). Controls showed a significant decrease in the amount of grey matter in the left posterior inferior frontal gyrus (Broca’s area) with age but there was no significant change observed in the amount of grey matter in this region across the age range in the group of PWS. These results taken together suggest that the normal patterns of maturation seen in the cortex are changed in PWS, potentially due to some experience-dependent plastic process. But, further studies using longitudinal measures are required to explore this further.

### 4.3 The relationship between stuttering severity and PT asymmetry

The significant findings in the original study (Foundas et al., 2004) claimed that the five PWS with the reversed (i.e. rightwards) asymmetry of the PT had the most severe stuttering and showed a significant improvement in stuttering under conditions of delayed auditory feedback. It should be noted, however, that although the nine PWS with typical leftwards asymmetry of the PT appeared unresponsive to feedback manipulations, the level of their stuttering was close to that of Controls and suggestive of a “floor” effect. In the current study, the relationship between stuttering severity and asymmetry in our group of 67 was examined and none was found; rightwards asymmetry was observed across the span of stuttering severity (see Fig. 5). Furthermore, the number of participants with rightwards asymmetry in the PWS group was approximately one-third in our group of 63 Controls and in the study of 14 PWS and 14 Controls (Foundas et al., 2004).

### 4.4 Relating structural asymmetry to functional lateralisation of language

Even though the conclusions of the Foundas et al. (2004) study are in accord with our current findings (i.e. no significant difference in asymmetry of the PT between PWS and Controls), the original study (Foundas et al., 2001) found a significant reduction in PT asymmetry in PWS and an overall larger size of this structure bilaterally (also not replicated in the present study). This work, along with the original theory put forward by Orton and Travis, contributed to the idea that PWS have altered asymmetry and specifically reduced functional lateralisation for language processing, which has persisted in the literature for a considerable time with some support (see recent work by Neef, Hoang, Neef, Paulus, & Sommer, 2015; Sato et al., 2011). The original claim (Orton, 1928) was that the two hemispheres might compete for “dominance” over language and affect speech behaviour in a deleterious fashion. The original basis for this hypothesis was the observed association with left-handedness and anecdotal reports that stuttering onset was often coincident with enforced use of the right hand (by parents, at school entry, or culturally). Orton also went on to conduct neurophysiological experiments recording “action currents” of the muscles in the left and right hands: During simultaneous movements, the action currents arrived predominantly at the right hand first in fluent speakers but at the left hand or coincidently in the two hands in a group of 17 PWS (Orton, 1928). Using modern techniques to measure cortical excitability during initiation of speech-related tongue movements, it has been demonstrated that PWS do not show the normal left-sided increase in excitability in the motor cortical representation of the tongue (Neef et al., 2015).

Functional imaging studies of stuttering reveal reduced lateralisation during language tasks. However, this is predominantly driven by an increase in right-hemisphere involvement rather than reduced or a lack of left-hemisphere activity. Recently, activity in the right-hemisphere homologue of Broca’s area (inferior frontal cortex) has been ascribed to an inhibitory response involved in stopping speech, that may be overactive when there is stuttering or a response to the urge to stutter (Neef et al., 2016).

The best evidence for altered functional lateralisation for language processing in stuttering comes from a study using near-infrared spectroscopy (Sato et al., 2011). Pre-school and school-aged children and adults who stutter listened passively to speech stimuli that differed either in the final phoneme or in prosody. Control children showed the expected left-lateralised blood flow response for the phoneme change and the right-lateralised response for the prosodic variation. Children and adults who stutter either showed no clear lateralised response or the reversed pattern in each condition. This abnormal pattern of asymmetry is present early in development and persists into adulthood. It is difficult to argue, therefore, that this pattern is a consequence of stuttering.

Despite this recent and convincing evidence for altered functional lateralisation of some aspects of speech and language processing in stuttering, it is important to consider how it relates to structural asymmetry such as asymmetry of the PT. In a study of adults who had evaluation of language dominance with the invasive sodium amobarbital test (one hemisphere is temporarily anesthetized while the other is tested) prior to epilepsy surgery, no relationship was found between language dominance and PT asymmetry (Dorsaint-Pierre et al., 2006). Instead, there was an unexpected rightwards asymmetry of the normally symmetric inferior frontal cortex (Broca’s area in the left hemisphere) in patients with right-hemisphere language dominance. On the basis of these findings, we suggest that the search for correlates of altered functional lateralisation in stuttering might focus on the anterior speech region in the frontal lobe rather than the posterior temporal cortex.

### 4.5 Limitations and future directions

The present study describes results obtained in a large sample of 67 people who stutter, spanning a wide age range from 12 to 54 years. However, we failed to replicate the previously described reduction in PT asymmetry noted in a group of 16 PWS with an average age of 31 years (Foundas et al., 2001). The PWS in that study also had a significantly larger PT bilaterally, which we also failed to replicate in our sample. It is worth noting, however, that even with our large sample, the size of the effect would need to be at least medium (0.5) to detect a significant (*p <* 0.05 two-tailed) difference between the two groups 90% of the time. Based on Fig. 3 in the previous report (Foundas et al., 2001), we estimate the effect size for the original finding to be very small (0.1). Furthermore, in our study, the datasets were acquired on different scanners at different field strengths, using different headcoils and sequences, which might have added extra variance and reduced power. It is worth noting that the datasets from the youngest participants were acquired on the 1.5T machines whereas the majority of the older participants were scanned at 3T. The largest subsample was obtained at 1.5T using a FLASH sequence in 30 PWS and 29 Controls. Analysis of the data obtained in this subsample resulted in the same failure to replicate a group difference in PT size and asymmetry. We conclude that if a significant difference in PT asymmetry exists in PWS it is of trivial effect size and requires substantial sample sizes to detect it. This then raises the question as to whether such a small but statistically significant difference has biological or clinical significance.

The significant group difference in the relationship with age and PT asymmetry (measured automatically using VBM), which was positive for Controls and not significant for PWS, could have been affected by a group of young controls scanned at 1.5T using MPRAGE. However, this result held when we excluded these datasets. Nevertheless, our findings of differences in the relationship of PT asymmetry and age in PWS compared with Controls warrant further investigations of different age groups. Furthermore, the gender differences observed for PT asymmetry in PWS require replication in a sample with a better sex ratio.

Finally, we note that it is possible that there are subtypes of individuals who stutter, who do so in relation to risk factors conferred by atypical brain anatomy either in the PT or in other cortical and subcortical structures (see Foundas, Mock, & Corey, 2013). Although our groups of people who stutter and controls showed very similar distributions of PT asymmetry, it is possible that within the stuttering group, an unusual asymmetry coupled with another (as yet unknown) factor would constitute sufficient risk to develop speech dysfluency.

## Conflict of interest

We wish to confirm that there are no known conflicts of interest associated with this publication and there has been no significant financial support for this work that could have influenced its outcome.
